# Case of Gun-Shot Wound of the Spine

**Published:** 1851-09

**Authors:** Charles S. Tripler

**Affiliations:** Surgeon U. S. Army


*Case of Gun-Shot Wound of the Spine.* By Charles S. Tripler, M.D., Surgeon U. S. Army.—The interesting and important discovery of the reflex function of the spinal nerves promises so much benefit to the science of medicine that no fact, tending to illustrate or establish Dr. Hall's views, can be looked upon with indifference. I therefore take great pleasure in communicating the following notes of an interesting case, which I made at the bedside of the patient a short time ago. I shall make no remarks upon it, preferring to submit it as it is, for the use of Dr. Hall himself, should it ever meet his eye. I

may, however, be indulged in suggesting that the practical surgeon may derive a hint from it that may save himself and his patient some trouble in a similar case; for, if the titillation of an afferent nerve may, through reflex action, enable him to dispense with the use of the catheter and enemata, it will be no trifling point gained.

During the protracted war with the Seminole Indians in Florida, an officer, traveling from St. Augustine to Picolata, was waylaid and wounded by a party of those savages. He was seated upon the floor of a common baggage wagon; the ball passed through the side of the vehicle before striking him. He was shot on the line of the union of the last dorsal, with the first lumbar vertebra—the ball penetrating at the angle of the ribs, on the right side, two inches above the vertebra, and passing in a direction obliquely downwards and toward the spine. The general direction of the wound was ascertained by the probe, but the ball could not be felt, and where it is lodged remains a mystery to this day.

This took place on the 25th November, 1839. The immediate consequences were loss of motion and sensation below the wounded part, though the sensorial recognition of the lower extremities was that of numbness and tumefaction. When he was received into the hospital, bottles of hot water were applied to his legs, with the effect of causing deep eschars very rapidly, but without producing any sensation. The gun-shot wound healed very readily, leaving the patient in the following condition:—The line of normal sensation began in front, at the anterior superior spinous process of the ilium, descended almost in the direction of Poupart's ligament about half its length, then curved upwards, passed just below the umbilicus, described a similar curve on the other side, and then passed around the back, in nearly a right line, to the point of departure.

The bladder and the rectum were paralyzed; the one was relieved by the catheter, the other by castor oil. The use of the oil was continued for about two years; afterwards, enemata were substituted; lavements of water are still used occasionally. The feces are passed without sensation. The catheter was used for about a year, or a little more. About the beginning of the year 1841, he found that the bladder could be induced to contract, by

*tickling the side of the penis, just behind the corona glandis;* and he afterwards discovered that the same manipulation would provoke the rectum to discharge its contents; no sensation, in the mean while, being transmitted to the sensorium.

He thinks that titillation of the left side of the penis affects the rectum more than the same operation upon the right.

No sensation of distended bladder calls for relief; but contraction of the toes, and abduction of both thighs, occur at this time, warning the patient of the wants of nature.

Priapism was readily excited, for a time, by friction upon the back or breast; but this seems to have subsided of late years.

The flexors of the toes are permanently about half contracted; by tickling or jerking up the scrotum and testicles, these muscles may be made to act spasmodically.

The temperature of the paralyzed parts is good. He thinks he feels more and more, from year to year, a consciousness of the existence of the limbs, and by an effort of the mind, to fix attention upon them, they ache so much as to render it necessary to desist.

There is not so much corpulency of body as is usual in such cases, nor are the paralyzed extremities so much atrophied as we might expect.

All sorts of counter-irritations, hydropathy, homœopathy, electricity, strychnia, &c., have been resorted to, but without benefit. In 1844 or '45, while trying the sulphur vapor, a jet of hot vapor was thrown upon the sole of the left foot, and took off the whole integument, he being totally unconscious of any sensation.

The urine was ammoniacal and purulent for the first three or four years, but has been less offensive since. If he assumes the erect position, leaning upon his crutches, to empty the bladder, the urine is less offensive than when he is obliged to lie in bed for a few days.

The color of the limbs is natural. He assures me that they were, a few years ago more sallow and more atrophied.—*New York Jour. of Med.*

